# Influence of invertor and evertor muscle fatigue on functional jump tests and postural control: A prospective cross-sectional study

**DOI:** 10.1016/j.clinsp.2022.100011

**Published:** 2022-02-26

**Authors:** Gabriela Borin Castillo, Guilherme Carlos Brech, Nátalia Mariana Silva Luna, Fernanda Botta Tarallo, Jose Maria Soares-Junior, Edmund Chada Baracat, Angelica Castilho Alonso, Júlia Maria D'Andréa Greve

**Affiliations:** a Laboratório de Estudos do Movimento, Instituto de Ortopedia e Traumatologia (IOT), Hospital das Clínicas HCFMUSP, Faculdade de Medicina, Universidade de São Paulo, São Paulo, SP, Brazil; b Programa de Pós-Graduação em Ciências do Envelhecimento, Universidade São Judas Tadeu (USJT), São Paulo, SP, Brazil; c Disciplina de Ginecologia, Departamento de Obstetrícia e Ginecologia, Hospital das Clínicas HCFMUSP, Faculdade de Medicina, Universidade de São Paulo, São Paulo, SP, Brazil

**Keywords:** Muscle fatigue, Ankle, Functional tests, Postural balance

## Abstract

**Objective:**

Fatigue of the ankle's stabilizing muscles may influence the performance of functional activities and postural control. The purpose of this study was to evaluate the performance of healthy young adults using functional jump tests and static posturography control under pre- and post-fatigue conditions of the ankle invertor and evertor muscles.

**Methods:**

Thirty physically active healthy male and female (15 male and 15 female) volunteers (24.3 years) were enrolled in this prospective cross-sectional study. Participants performed tests on one day under a non-fatigued state of invertor and evertor muscles and on the second day in a fatigued state. Tests included static posturography on a force platform in a bipedal stance with eyes open and closed and in one-legged support with eyes open and functional jump tests (figure-of-8, side hop, 6-m crossover hop, and square hop). Fatigue of the ankle invertor and evertor muscles was induced using isokinetic dynamometry with 30 repetitions at 120°/s.

**Results:**

Participants had an average age of 24.3 years (SD ± 2.08), the height of 1.73 m (SD ± 0.08), and a weight of 68.63 kg (SD ± 10.29). The average Body Mass Index (BMI) was 22.88 (SD ± 2.46). A decrease in performance was observed in functional activities and postural control under all conditions after the induction of muscle fatigue, except for the speed at a bipedal stance with eyes open.

**Conclusions:**

Functional jump tests are low cost and useful for clinical practice and evaluation of the effects of muscle fatigue and could be used in clinical practice.

## Highlights

• Fatigue of the ankle's stabilizing muscles does influence the performance.
• Functional jump tests are low cost and useful for clinical practice.
• Functional jump tests can evaluate of the effects of muscle fatigue.

## Introduction

The lateral ankle sprain is an injury that occurs when you roll, twist, or turn your ankle in an awkward way, it is one of the most common injuries in athletes and non-athletes and represents 15–20% of all sports injuries. Further, 20% of these patients can develop chronic ankle instability.^1^ A sprained ankle is directly related to the injury mechanism, and muscle fatigue is an indirect factor that leads to sprains by increasing the instability of the joint during sports practice or habitual gait.^2^

Fatigue of the ankle invertor and evertor muscles may decrease the power and speed of sensorimotor responses, as well as the power of muscle response, all factors that contribute to increased joint instability.^2^^,^^3^ Moreover, fatigue slows reaction time by decreasing the activity of muscle receptors and changing the neuromuscular system's stability control. Muscle fatigue is responsible for many athletic injuries, especially toward the end of an activity, probably as a result of functional instability of the joint.^2^^,^^3^

Different methods exist for evaluating the influence of muscle fatigue on neuromuscular control. Functional evaluation of the ankle joint through the implementation of tasks can be useful for understanding the mechanism of maintaining stability, as well as the effects of muscle fatigue. Professionals can use this kind of evaluation as a tool to assist in prevention, treatment, and return to sport for athletes with ankle injuries.^4^

The functional performance tests simulate the conditions of the sport practiced and are commonly used to objectively assess the athlete's progress in rehabilitation and the safety to return to practice after the injury.^5^ Previously, four proposed tests (figure-of-8, side hop, 6-m crossover hop, and square hop) were effective and simulated the movements and forces imposed on the ankle joint while performing an activity.^6^

Static posturography on a force platform is a tool that evaluates postural control by measuring the displacement of the Center of Pressure (COP).^7^ Some authors suggest that a greater displacement of COP indicates functional postural instability that may be related to sporting performance and injury risk.^8^ The lack of more specific and dynamic tests as discharge criteria and treatment progress reviews often leads professionals to work empirically. The establishment of more specific evaluations could improve the understanding of the influence of fatigue on ankle stability. Moreover, static posturography is a very useful and inexpensive tool for clinical practice and can be used in injury prevention, training level determination, injury rehabilitation, and the safe return to sporting activities. The authors’ hypothesis is that fatigue of the invertor and evertor ankle muscles could be worst for motor control. The objective of this study was to evaluate the performance of healthy young adults during functional tests and static posturography under pre- and post-fatigue conditions of the invertor and evertor ankle muscles.

## Methods

### Study design, setting, sample size

This prospective cross-sectional study included 30 healthy (15 male and 15 female) volunteers. For the size of the sample calculation, the authors assume the two-tailed hypothesis: alpha values (probability of error type 1) of 5%, beta values (probability of error type 2) of 10%, and test strength of 90%. According to this sample size calculation, a sample of 26 individuals gave a significance level of 5%. All participants were informed about the procedures and signed an informed consent form. Participants answered the question, “Which is your favorite leg for kicking a ball?” to define the dominance of the lower limbs. In this study, 20 participants had right dominance, and 10 had left dominance. The frequency of physical activity was 2h/week (53.3%) or 3h/week (46.6%) with an average frequency of 1.75h/day (SD ± 0.64).

### Participant

Volunteers were recruited from sporting clubs in the city of São Paulo. Participants were included if they had performed physical activity at least twice a week for three months prior to the test.^9^ Individuals were excluded if they had experienced an injury in the lower limb joints in the past six months, with injury defined as an event in which the individual did not practice physical activity for > 24 consecutive hours. They were also excluded if they felt any pain during the tests, had a history of injury or surgery of the hip, knee, and/or ankle, or had a history of neurological, cardiovascular, metabolic, rheumatic, and/or vestibular system disorders.

### Measurements

The participants performed two evaluation sessions at an interval of at least 48h, the same researcher selected and evaluated the participants (was not blind, the same research enrolled and assigned the participants). On the first day, they performed posturography and functional tests in the non-fatigued condition. On the second day, after inducing fatigue of the ankle invertor and evertor muscles using an isokinetic dynamometer (Biodex Medical Systems, System 3, Software version 3.2), the volunteers performed the same tests as on the first day. Each participant was exposed to an experimentally random order. Before each test, participants performed a 5-min warm-up on a stationary bike with submaximal effort (load and comfortable cadence, without fatigue). In order to induce muscle fatigue, participants sat with the limb to be tested supported at the distal thigh and their foot resting on a rigid plate in neutral. Thirty concentric repetitions of ankle inversion and eversion were performed by each participant at a speed of 120°/s by each participant.

### Posturography

The postural balance assessment (posturography) was performed on a portable force platform (AccuSway Plus, AMTI®, MA, USA). For data acquisition, the force platform was connected to a signal-amplifying interface box (PJB-101) linked to a computer using an RS-232 cable. The data were gathered and stored using Balance Clinic® software, configured to a frequency of 100 Hz with a fourth-order Butterworth filter and a cutoff frequency of 10 Hz. All subjects underwent the test with standardized positioning in relation to the maximum width of the support base (smaller than hip-width), with arms along the body and head looking straight at a target. The base of support was drawn on paper in a fixed position on the force platform, corresponding to the anatomical points of the distal hallux phalanx, fifth metatarsal head, and lateral and medial malleolus for each foot.^7^

Three measurements were made with the Eyes Open (EO) and three with the Eyes Closed (EC) for 60 seconds each. Subsequently, three measurements were performed by each participant on one foot with EO for 30 seconds each. In this condition, participants were instructed by the researcher to maintain a 90° knee flexion with the contralateral leg and neutral hips with arms along the body while looking at the same fixed point. The mean of the results was calculated from the three tests conducted under each condition and was processed using Balance Clinic® software. The parameters used to measure the subject's stability with eyes open and closed were the root mean square of the displacement from the COP in the Mediolateral Axis (XSD) and Anteroposterior Axis (YSD), the mean velocity calculated from the total displacement of the COP in all directions (VAvg), and the elliptical displacement Area 95% (Area95).^7^

### Functional hop tests

This section consisted of four functional tests: figure-of-8, side hop, 6-m crossover hop, and square hop.^10^ The authors timed each test using an electronic timer. All participants performed up to three practice trials of each performance test to familiarize themselves with the testing procedures, followed by three trials at maximal effort. The participants rested for at least one minute between each individual trial. The authors counterbalanced the order of functional tests and limbs for all participants. Each test was performed by hopping on one limb as quickly as possible. The authors also marked any trials in which a participant put the contralateral foot down, fell, or did not complete the course as outlined, and they were asked to perform the trial again.

For the figure-of-8 test, a 5-m course outlined by the cones was used. Each participant was instructed to hop twice around the course. For the side hop test, participants hopped laterally over a 30 cm distance. One repetition constituted hopping laterally 30 cm and back to the starting point, completing 10 repetitions. In the 6-m crossover hop test, a 6-m long line was used. The participants were instructed to hop diagonally over the 15 cm-wide line, alternating sides for the entire 6-m. The square hop test consisted of a 40 × 40 cm square marked on the floor with tape. Starting outside the square, the participants were instructed to hop in and out of the square for five repetitions. One repetition constituted hopping in and out of the tape, circulating completely around the square and back to the starting point. With the right limb, the participants hopped in a clockwise direction, and with the left limb, they hopped in a counterclockwise direction.

### Ethics

The study was conducted at the Institute of Orthopedics and Traumatology, Hospital das Clinicas, School of Medicine, University of São Paulo, with approval granted by the Ethics Committee of the University of São Paulo (CaPPesq n° 267/12). All participants signed the informed consent form.

### Statistical analysis

All variables are displayed in the tables. Descriptive statistics included calculating the mean, standard deviation, minimum and maximum values for quantitative variables, and absolute and relative frequencies for categorical variables.

Posturography for the four variables and functional jump tests under pre- and post-fatigue conditions were analyzed using the Shapiro-Wilk test of normality. The Wilcoxon signed-rank test was used for the analysis of the continuous variables of posturography and functional jump tests.

Analyses were performed using the statistical software SPSS (Statistical Package for Social Science) version 15.0 for Windows, and the value of p≤0.05 was considered statistically significant**.**

## Results

Participants had an average age of 24.3 years (SD ± 2.08), a height of 1.73 m (SD ± 0.08), and a weight of 68.63 kg (SD ± 10.29). The average Body Mass Index (BMI) was 22.88 (SD ± 2.46). The baseline characteristics of the groups are shown in Table 1.

**Table 1:** Baseline characteristics of the groups.

Variable (n = 30)	Average
Age	24.3 years (± 2.08)
Height	1.73 m (± 0.08)
Weight	68.63 kg (± 10.29)
Body mass index	22.88 (± 2.46)

Data from the force platform revealed the following results for pre- and post-fatigue. In the bipedal stance with eyes open, there was a statistical difference among XSD and YSD displacement from the COP, and elliptical displacement Area 95%. In the bipedal stance with the eyes closed, there were statistical differences for all variables (Table 2).

**Table 2:** Static posturography pre and post invertor and evertor muscle fatigue: open and closed eyes in bipedal stance.

Variable	PrePost		P	PrePost		p
	Open Eyes			Closed Eyes		
XSD	0.23	0.32	**< 0.001**^a^	0.30	0.36	**< 0.001**^a^
YSD	0.44	0.59	**< 0.001**^a^	0.48	0.65	**< 0.001**^a^
VAvg	0.82	0.92	0.168	1.14	1.81	**< 0.001**^a^
Area 95	1.97	2.61	**< 0.001**^a^	2.74	3.97	**< 0.001**^a^

a Wilcoxon, p ≤ 0.05. XSD, Mediolateral displacement from the COP (cm); YSD, Anteroposterior displacement from the COP (cm); VAvg, Mean velocity calculated from the total displacement of the COP in all directions (cm/s) and Area 95, Elliptical displacement area 95% (cm^2^).

In both the right and left unipedal stances with the EO, there was a statistical difference for all variables (Table 3).

**Table 3:** Static posturography pre and post invertor and evertor muscle fatigue with open eyes in unipedal stance.

Variable	PrePost		P	PrePost		p
	Unipedal stance right			Unipedal stance left		
XSD	0.61	0.78	**< 0.001**^a^	0.53	0.79	**< 0.001**^a^
YSD	0.69	0.89	**< 0.001**^a^	0.72	0.89	**< 0.001**^a^
VAvg	4.01	4.78	**< 0.001**^a^	3.8	4.21	**< 0.001**^a^
Area 95	6.32	9.07	**< 0.001**^a^	6.32	8.14	**< 0.001**^a^

a Wilcoxon, p ≤ 0.05. XSD, Mediolateral displacement from the COP (cm); YSD, Anteroposterior displacement from the COP (cm); VAvg, Mean velocity calculated from the total displacement of the COP in all directions (cm/s) and Area 95, Elliptical displacement area 95% (cm^2^).

All functional tests showed significant statistical differences between pre- and post-fatigue conditions (Table 4).

**Table 4:** Functional performance functional jump tests (time in seconds).

Variable	Pre	Post	p
Square right	23.67	31.93	**< 0.001**^a^
Square left	23.32	31.6	**< 0.001**^a^
Cross right	3.32	5.62	**< 0.001**^a^
Cross left	3.45	5.75	**< 0.001**^a^
Lateral right	10.58	15.61	**< 0.001**^a^
Lateral left	10.45	15.82	**< 0.001**^a^
Eight right	11.31	15.71	**< 0.001**^a^
Eight left	11.39	15.72	**< 0.001**^a^

a Wilcoxon, p ≤ 0.05.

## Discussion

Fatigue of the ankle invertor and evertor muscles was found to influence performance in functional tests and posturography. The results revealed an increase in posturography oscillation, speed, area of COP displacement, and time required for functional tests.

One goal of the present study was to observe the influence of fatigue on postural control. In agreement with other results, the authors found that muscle fatigue alters postural control, increasing the speed and amplitude of displacement in young adults,^11^ and these changes may be exacerbated due to an additive effect of fatigue and a change in the sense of joint position and strength.^12^

Moreover, a systematic review^11^ showed several studies that claimed that local short exercises could induce deficits in postural control when the loss in maximum voluntary contraction was ≥30%. In the present study, the fatigue index obtained using the isokinetic dynamometer showed values above 30%, corroborating the data cited in the literature.

The results of this study suggest a relationship between task difficulty and the relevance of visual information and support base. In the bipedal stance with EO, there was no observed difference in the speed variable. Moreover, in comparison with variables under other conditions, lower values were observed. This can be explained by the less challenging situations for the group of participants evaluated, as they were young and active without any injury, which is consistent with the literature.^13^

Indeed, some authors have demonstrated modulation of the effect of fatigue on postural control according to the source of sensory information available, such as EO and EC. In the present study, the bipedal stance with EC had a difference in all variables, including speed. The withdrawal of visual ability demanded greater involvement of the neuromuscular system, thereby resulting in an increase in the speed of COP displacement to maintain it within the limits of stability.^14^ The afferent sensorimotor information was reduced with muscle fatigue, and vision was even more important for postural balance adjustments.^15^ In both the right and left one-legged support with EO, there was increased mediolateral and anteroposterior sway and Area95, as well as speed. Some authors^13^, ^14^, ^15^, ^16^ argue that a small support base (single leg) demands greater activity of the tonic postural muscles and postural adjustment to maintain balance, and this is independent of fatigue. However, there was an association between leg support and fatigue, which caused a further increase in COP displacement and speed, demonstrating the importance of fatigue in the mechanisms of dynamic stability of the ankle.

The results of the present study indicate that fatigue in the distal muscles of the lower extremities is associated with deficits in postural control in the sagittal and frontal planes. These results are consistent with one study that found increased postural control in both the sagittal and frontal planes after evertor and invertor fatigue.^15^ However, it was contradictory with another study that reported differences in COP displacement only in the sagittal plane;^17^ this could be explained by the fact that their study induced fatigue in different muscles (the plantar flexors and dorsiflexors).

The selection of the four functional jump tests used in this study was based on the ability to assess the stability and lateral structure of the ankle joint based on the movements required for cuts and turns. These jump tests are reproducible and reliable.^18^ Further, the lateral jump test, in particular, requires a broad movement of inversion and eversion in dynamic situations and can reveal a deficiency of the fibular and tibial muscles in the frontal plane.^19^^,^^20^ The muscle peroneus longus remained active during all phases of the lateral jump test, and thus, the increase in execution time of this specific test could be related to its dysfunction.^20^

In the present study, the authors observed that after inducing fatigue of the invertors and evertors, there was a decrease in dynamic stabilization, seen with longer performance in the four functional tests. Invertor and evertor muscles are mediolateral stabilizers of the ankle and play a fundamental role in dynamic stabilization.^21^ Their fatigue affects proprioception and kinesthetics of the ankle joint by increasing the threshold for discharge of muscle spindle afference and a decreased sense of alertness, thereby decreasing performance in dynamic activities and predisposing to injuries.^22^^,^^23^

Rehabilitation and return to activity after injury are directly related to the responsiveness of the muscles and joint structures involved. Data from the present study have opened the possibility of studying electromyographic activity during the execution of functional jump tests and posturography, thus contributing to a greater definition of the action of different muscles during tasks.

Furthermore, quantitative tests that help to functionally evaluate individuals for training and treatment are critical for decision-making in clinical practice. Assessment of joint function and postural control can improve the quality of training or treatment and performance, preventing the early return to the sport and the risk of recurrent lesions.^23^ Functional jump tests used in this study are low cost, easy to apply, and sensitive enough to evaluate the effect of muscle fatigue in healthy subjects. The study results may have a positive influence on the scientific and clinical aspect as it allows us to understand that many injuries are caused by fatigue, therefore, it would be possible to prevent them.

## Conclusion

Fatigue of the ankle invertor and evertor muscles in physically active young individuals reduced their performance in four functional jump tests and increased proprioceptive activity for postural control in static posturography.

## CRediT authorship contribution statement

**Gabriela Borin Castillo:** Investigation, Writing – original draft. **Guilherme Carlos Brech:** Investigation, Writing – review & editing. **Nátalia Mariana Silva Luna:** Investigation, Writing – original draft. **Fernanda Botta Tarallo:** Investigation, Writing – original draft. **Jose Maria Soares-Junior:** Investigation, Writing – review & editing. **Edmund Chada Baracat:** Investigation, Writing – review & editing. **Angelica Castilho Alonso:** Formal analysis, Writing – review & editing. **Júlia Maria D'Andréa Greve:** Supervision, Writing – review & editing.

## Conflicts of interest

The authors declare no conflicts of interest.
